# Veno-venous extracorporeal membrane oxygenation for perioperative management of infective endocarditis after COVID-19 with acute respiratory distress syndrome: a case report

**DOI:** 10.1186/s13019-024-02890-w

**Published:** 2024-06-24

**Authors:** Ken Sawada, Takahiro Kawaji, Koji Yamana, Kazuki Matsuhashi, Yoshitaka Hara, Naohide Kuriyama, Tomoyuki Nakamura, Atsuo Maekawa, Yasushi Takagi, Osamu Nishida

**Affiliations:** 1 Department of Anesthesiology and Critical Care Medicine, School of Medicine, Fujita Health University, 1-98 Dengakugakubo, Kutsukake-cho, Toyoake, Aichi 470-1192 Japan; 2 Department of Cardiovascular Surgery, School of Medicine, Fujita Health University, Toyoake, Aichi Japan

**Keywords:** Infective endocarditis, COVID-19, Acute respiratory distress syndrome, Veno-venous extracorporeal membrane oxygenation, Pulmonary damage

## Abstract

**Background:**

Infective endocarditis (IE) is a rare cardiovascular complication in patients with coronavirus disease 2019 (COVID-19). IE after COVID-19 can also be complicated by acute respiratory distress syndrome (ARDS); however, the guidelines for the treatment of such cases are not clear. Here, we report a case of perioperative management of post-COVID-19 IE with ARDS using veno-venous extracorporeal membrane oxygenation (V-V ECMO).

**Case presentation:**

The patient was a 40-year-old woman who was admitted on day 18 of COVID-19 onset and was administered oxygen therapy, remdesivir, and dexamethasone. The patient’s condition improved; however, on day 24 of hospitalization, the patient developed hypoxemia and was admitted to the intensive care unit (ICU) due to respiratory failure. Blood culture revealed *Corynebacterium striatum*, and transesophageal echocardiography revealed vegetation on the aortic and mitral valves. Valve destruction was mild, and the cause of respiratory failure was thought to be ARDS. Despite continued antimicrobial therapy, ARDS did not improve the patient’s condition, and valve destruction progressed; therefore, surgical treatment was scheduled on day 13 of ICU admission. After preoperative consultation with the team, a decision was made to initiate V-V ECMO after the patient was weaned from CPB, with concerns about further worsening of her respiratory status after surgery. The patient returned to the ICU with transition to V-V ECMO, and her circulation remained stable. The patient was weaned off V-V ECMO on postoperative day 33 and discharged from the ICU on postoperative day 47.

**Conclusions:**

ARDS may occur in patients with IE after COVID-19. Owing to concerns about further exacerbation of pulmonary damage, the timing of surgery should be comprehensively considered. Preoperatively, clinicians should discuss perioperative ECMO introduction and configuration.

## Background

Infective endocarditis (IE) is a rare cardiovascular complication in patients with COVID-19 [1], the number of reported cases has increased recently [2, 3]. Coinfection is usually diagnosed during the course of COVID-19; however, IE may occasionally be detected after COVID-19. In patients with IE after COVID-19, the causes of respiratory failure include heart failure, septic acute respiratory distress syndrome (ARDS), secondary bacterial pneumonia [4], or COVID-19 recurrence [5]. However, lung injury caused by surgical procedures [6] and cardiopulmonary bypass (CPB) [7] should be considered before indicating surgery in patients with IE after COVID-19. Furthermore, postoperative pulmonary complications often occur in patients who undergo surgery for perioperative COVID-19 [8]. Therefore, deciding the timing of surgery and perioperative management is challenging, especially without published guidelines. Recently, extracorporeal mechanical support devices have become essential for the perioperative management of critically ill patients [9]. Herein, we report a case in which veno-venous extracorporeal membrane oxygenation (V-V ECMO) was used for the perioperative management of IE after COVID-19 with ARDS.

## Case presentation

**Fig. 1:**
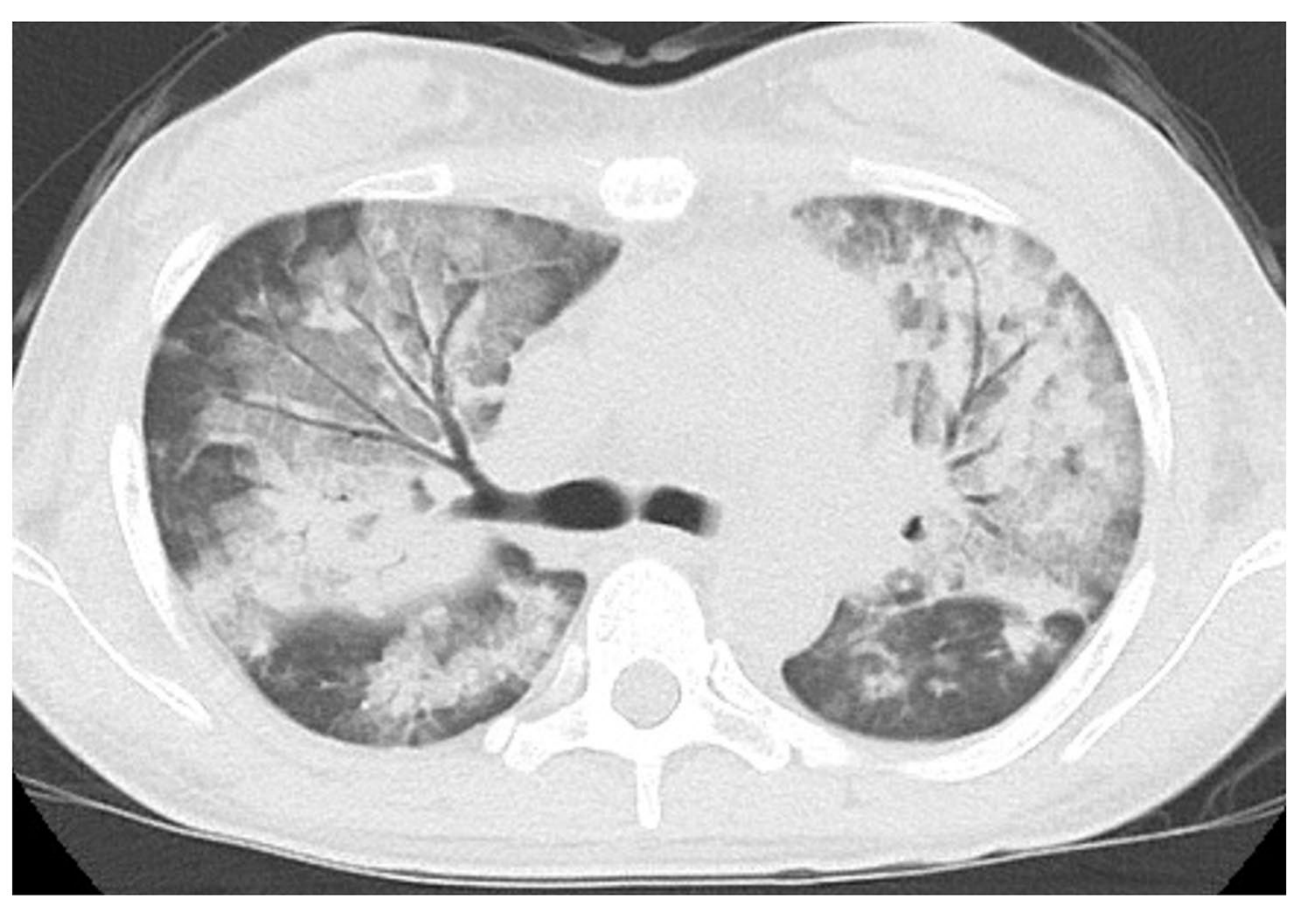
Computed tomography on admission: multiple frosted shadows are seen in the bilateral lung fields

**Fig. 2:**
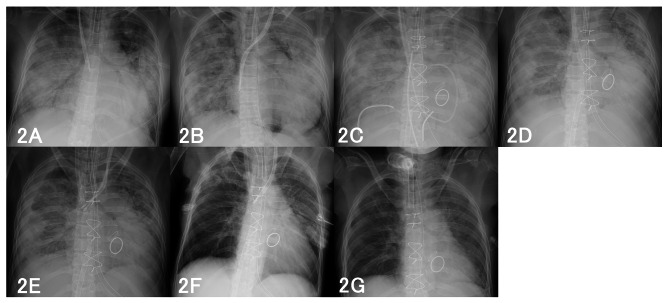
Chest radiographs during the course of treatment. **A**: on admission to ICU, **B**: preoperative radiograph, **C**: postoperative radiograph, **D**: before prone positioning, **E**: after prone positioning, **F**: before extracorporeal membrane oxygenation (ECMO) withdrawal, **G**: after respiratory weaning

**Fig. 3:**
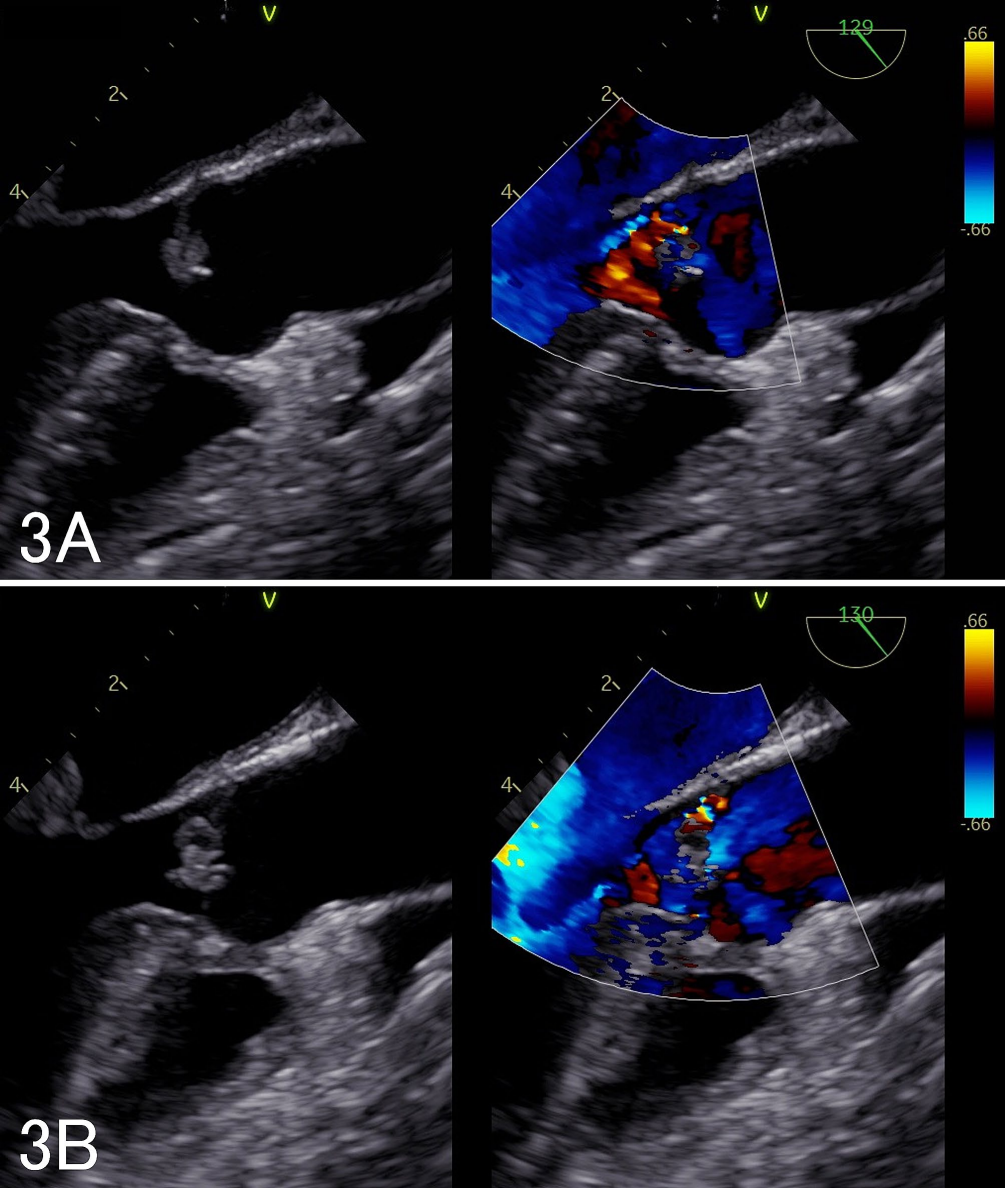
Comparison of aortic valve long-axis images. **A**: Post-ICU admission transesophageal echocardiography (TEE): verrucae adhered to the aortic valve and aortic regurgitation were observed. **B**: Preoperative TEE showing enlarged verrucae and worsened aortic regurgitation

A 40-year-old woman (height, 156 cm; body weight, 43 kg; body surface area, 1.38 m^2^) had undergone simultaneous pancreas-kidney transplantation for type 1 diabetes mellitus 8 years previously and had been receiving immunosuppressive therapy using prednisolone, tacrolimus, and everolimus. The patient was diagnosed with COVID-19 in September 2022; on the 18th day after disease onset, the patient was admitted to our hospital owing of hypoxia and worsening general malaise. Computed tomography (CT) showed ground-glass opacities in bilateral lungs, and oxygen, remdesivir, and dexamethasone were administered. On the 14th day of hospitalization, the patient did not require oxygen therapy and showed improved ground-glass opacities in the bilateral lungs. However, since she still had a slight fever and forearm and hand pain, a bacterial infection was suspected. On the 24th day of hospitalization, the patient developed hypoxemia, and CT revealed ground-glass opacities in both lungs again (Fig. 1). The patient’s respiratory condition rapidly deteriorated, and she was intubated in the intensive care unit (ICU). Her arterial blood gas (ABG) showed the following: pH 7.42, PaCO_2_ 37 mmHg, PaO_2_ 79 mmHg on pressure-controlled ventilation (PCV), fraction of inspiratory oxygen (F_I_O_2_) 0.35, pressure control (PC) 15 cmH_2_O, positive end expiratory pressure (PEEP) 10 cmH_2_O, and respiration rate (RR) 15/min. The PaO_2_/F_I_O_2_ ratio (PFR) was 201, and compliance of the total respiratory system (Crs) was 21 mL/cmH_2_O. Several chest radiographs within the ICU stay are shown in Fig. 2. Blood tests on ICU admission revealed the following results: white blood cell count, 21,000/µL; platelet count, 4.9 × 10^4^/µL; blood urea nitrogen level, 106 mg/dL; creatinine level, 4.6 mg/dL; C-reactive protein level, 22 mg/dL; and procalcitonin level, 1.3 mg/dL. Severe acute respiratory syndrome coronavirus 2 (SARS-CoV-2) antigen test results were negative. Piperacillin-tazobactam were administered as empirical antibiotics to assess bacterial infection. Immunosuppressive therapy after simultaneous pancreas-kidney transplantation was continued combined with only steroids concerning that bacterial infection would worsen. As the patient had anuria, kidney replacement therapy was administered for fluid correction to treat overhydration, thereby, hemodynamics was maintained. Although the etiologies of anuria might have included sepsis, side effects of medication, and acute rejection, none could be identified. On the 2nd day in the ICU, *Corynebacterium striatum* was detected in blood cultures, and vancomycin was administered. Transthoracic echocardiography showed mitral valve thickening, suspecting vegetation. On the 4th day in the ICU, transesophageal echocardiography (TEE) revealed vegetation on the aortic (Fig. 3A) and mitral valves, leading to a diagnosis of IE. All aortic valve leaflets had vegetation length of 4 mm. The mitral valve showed three contiguous papillary, irregularly shaped, and mobile vegetations extending 5–15 mm from the entire A2P2 junction. Valve dysfunction was mild, and the ejection fraction was normal. As the patient’s cardiac function was normal, ARDS was suspected to be the cause of respiratory failure rather than heart failure. After discussing the timing of the surgery with the team, it was decided to continue antimicrobial therapy to improve her general condition. Blood cultures were negative; however, the patient’s pulmonary function did not improve. The Crs of the patient’s lungs decreased to 15 mL/cmH_2_O, resulting in hypercapnia, and chest radiography showed mediastinal emphysema. Regarding mediastinal emphysema, we avoided excessive ventilation pressures using mechanical ventilation. Although we applied the prone position, it was discontinued due to hemodynamic instability. On the 11th day in the ICU, a follow-up TEE revealed progressive aortic valve regurgitation (Fig. 3B). The patient was scheduled to undergo aortic and mitral valve replacement and the removal of vegetation that causes of septic ARDS.

After preoperative consultation with the team, a decision was made to initiate V-V ECMO after the patient was weaned from CPB, with concerns about further worsening of her respiratory status after surgery. The patient was transported from the ICU to the operating room under intubation and sedation. Hemodynamic evaluation was conducted via TEE and a Swan-Ganz pulmonary artery catheter. During induction of general anesthesia, anesthesiologists inserted a drainage cannula (19-Fr cannula for drainage, HLS Cannula®; Getinge, Gothenburg, Sweden) into the right atrium via the right internal jugular vein. After the loss of spontaneous breathing, mechanical ventilation could not provide effective ventilation and acidemia progressed rapidly. The patient’s hemodynamics became unstable because of acidemia, and surgery was promptly initiated. An inflow cannula was inserted into the ascending aorta, and a drainage cannula (23-Fr cannula for drainage, Bio-Medicus® Nextgen; Medtronic, Minneapolis, MN, USA) was inserted into the inferior vena cava via the right femoral vein to establish CPB. Aortic and mitral valve replacement was performed with mechanical valve in consideration of life expectancy. After valve replacement, TEE showed no abnormalities in the replaced valve; circulation was maintained with inotropic support at the time of weaning from the CPB. We temporarily stopped the pump and assessed ABG while the cannula remained in position without protamine infusion. ABG test showed severe hypoxemia (PFR, 70) and respiratory acidosis due to which there was a failure of ventilation and accumulation of carbon dioxide. Hemodynamic parameters rapidly deteriorated and the pump was restarted. Since the cause of hemodynamic instability was poor ventilation, we decided to initiate V-V ECMO. Therefore, the perfusionist primed ECMO circuit and the patient was promptly transitioned from CPB to V-V ECMO (Cardiohelp System; Getinge). A drainage cannula in the patient’s right internal jugular vein was used as the return cannula to establish V-V ECMO. After V-V ECMO administration, the patient’s hemodynamics stabilized. Protamine was administered to neutralize heparin after V-V ECMO conversion. Thereafter, the chest was closed. The patient was returned to the ICU under sedative intubation.

In the ICU, the ECMO settings were 2700 rpm, flow rate at 3 L/min, and a sweep gas flow of 3.5 L/min. Ventilation was set at PCV, F_I_O_2_ 0.3, PC 5 cmH_2_O, PEEP 7 cmH_2_O, and RR 6/min. The peripheral capillary oxygen saturation (SpO_2_) was kept at > 95%. A heparin drip was used to prevent blood clots. On postoperative day 6, a brain CT scan revealed scattered subcortical brain hemorrhages complicating IE. After consultation with a neurosurgeon, it was determined that anticoagulation could be continued because the cerebral hemorrhage was mild. Originally targeting an activated partial thromboplastin time (aPTT) of 60–80 s in the patient receiving ECMO support, after cerebral hemorrhage we targeted the aPTT range of 60–70 s. Despite daily bronchoscopic clearance of the sputum, white viscous sputum obstructed the patient’s bilateral bronchi. For sputum excretion, prone positioning was initiated on postoperative day 12. Tracheostomy was performed on postoperative day 18. Chest radiography showed improvement in the lung field and Crs. On postoperative day 20, the patient exhibited no abnormal neurological findings, and her lung function improved (PFR: 493 and Crs: 30 mL/cmH_2_0). Therefore, V-V ECMO was discontinued on postoperative day 33. Mechanical ventilation was discontinued on day 35 and the patient was discharged from the ICU on postoperative day 47. Function of the transplanted organ was preserved, and blood purification and insulin therapy were weaned. The patient was discharged without assistance on postoperative day 107.

## Discussion

Reports of hospital-onset IE increased during the COVID-19 pandemic [9]. IE occurs in approximately 0.1% of patients hospitalized with COVID-19 [1]. IE and COVID-19 may be initially difficult to distinguish owing to their similar presentations. Therefore, the co-occurrence of IE and COVID-19 may lead to a delayed diagnosis, and IE is sometimes diagnosed after COVID-19. IE in patients with solid organ transplantation is often a nosocomial infection, and more than 70% of cases are considered to be hospital- and healthcare-related [10]. The most common causative organisms of IE in patients with COVID-19 are *Staphylococcus aureus* and *Enterococcus faecalis* [2, 3]; however, the causative organisms of IE in solid organ transplant recipients are diverse, with reports of *C. striatum* [11]. *C. striatum* is a low-virulence organism that generally colonizes healthy skin and is associated with nosocomial infections, particularly in immunosuppressed patients. In this case, there was no persistent fever or other signs before COVID-19, and IE was diagnosed after the course of COVID-19. Our patient exhibited several factors associated with IE, including post-transplant immunosuppression, immunosuppression caused by medications used to treat COVID-19, and the potential risk of catheter-related bloodstream infections. SARS-CoV-2 causes severe injury to the alveolar epithelium [12] and vascular endothelium [13]. In the present case, it is likely that COVID-19 caused severe alveolar inflammation, which triggered systemic inflammation due to sepsis, resulting in mixed severe inflammation, leading to the development of ARDS [14].

Current indications for surgical treatment of IE are as follows: (1) refractory heart failure directly related to valve dysfunction, (2) uncontrolled infection, and (3) prevention of embolic phenomena [15]. However, if the lung damage is severe in IE complicated by ARDS, decision-making regarding the timing of surgery and postoperative management can be difficult, and there are currently no clear treatment guidelines. In addition, there are reports of respiratory deterioration triggered by general anesthesia after COVID-19, and it is recommended that even standby surgery be delayed for 7 weeks [16]. In our case, the initial evaluation revealed mild valve destruction, and medical therapy was preferred in view of the post-COVID-19 infection, surgical invasiveness, and concerns about lung damage from CPB. Subsequently, the patient was switched to a surgical strategy owing to progressive valve destruction and worsening lung damage despite effective antimicrobial therapy. The benefits of early or late surgical intervention have been debated extensively; however, solid supporting evidence is lacking. Therefore, a comprehensive team decision must be made on an individual, case-by-case basis. When considering surgery, it is necessary to anticipate the exacerbation of lung damage. When ARDS develops after cardiac surgery with CPB, V-V ECMO has been reported as a bridge to CPB [17]. In the present case, preoperative complications of ARDS prompted preoperative considerations regarding configurations, such as the CPB circuit and cannula size, in anticipation of postoperative V-V ECMO management. CPB was established by inserting drainage cannulas from the right internal jugular and right femoral veins and inserting a reinfusion cannula into the ascending aorta. Using the cannula via the internal jugular vein as the reinfusion cannula for the two drainage cannulas used in CPB, the transition from CPB to V-V ECMO was smooth. Stable postoperative ECMO management was achieved by selecting an appropriately sized cannula to obtain stable ECMO blood flow during long-term management. To the best of our knowledge, this is the first report of successful perioperative ECMO for ARDS in patients with IE after COVID-19.

Prone positioning is an option for lung-protective ventilation in ARDS [18]. Prone positioning after open-heart surgery is concerned about wound infection and hemodynamic instability. However, the effects on the respiratory status and safety of prone positioning for respiratory failure after open-heart surgery and its effects on the wound and hemodynamics have been reported [19, 20]. In our case, prone positioning was used in combination with ECMO management without any complications. There were also concerns about the effect of prone positioning on abdominal compression on the transplanted organ; however, the graft improved without malfunction.

## Conclusion

ARDS may occur in patients with IE after COVID-19. To avoid further exacerbation of pulmonary damage, the timing of surgery should be comprehensively considered. Preoperatively, clinicians should discuss perioperative ECMO introduction and configuration.

## Data Availability

No datasets were generated or analysed during the current study.

## Abbreviations

aPTT: activated partial thromboplastin time
ARDS: acute respiratory distress syndrome
COVID-19: coronavirus disease 2019
IE: infective endocarditis
CT: computed tomography
ICU: intensive care unit
PCV: pressure-controlled ventilation
FIO2: fraction of inspiratory oxygen
PC: pressure control
PEEP: positive end expiratory pressure
RR: respiration rate
PFR: PaO2/FIO2 ratio
Crs: compliance of the total respiratory system
TEE: transesophageal echocardiography
V-V ECMO: veno-venous extracorporeal membrane oxygenation
